# Angiofibroma Originating outside the Nasopharynx: A Management Dilemma

**DOI:** 10.1155/2016/3065657

**Published:** 2016-11-13

**Authors:** Ashraf Nabeel Mahmood, Rashid Sheikh, Hamad Al Saey, Sarah Ashkanani, Shanmugam Ganesan

**Affiliations:** ^1^Rhinology Section, Otorhinolaryngology, Head & Neck Surgery (ORL-HNS) Department, Rumailah Hospital, Hamad Medical Corporation, Doha, Qatar; ^2^Otorhinolaryngology, Head & Neck Surgery (ORL-HNS) Department, Rumailah Hospital, Hamad Medical Corporation, Doha, Qatar; ^3^Weill Cornell Medical College, Ar-Rayyan, Qatar

## Abstract

*Background*. Angiofibroma is a benign tumor, consisting of fibrous tissue with varying degrees of vascularity, characterized by proliferation of stellate and spindle cells around the blood vessels. It most commonly arises from the nasopharynx, although it may rarely arise in extranasopharyngeal sites.* Case Report*. A 46-year-old male presented with left side nasal obstruction and epistaxis for one month. Clinical nasal examination revealed left sided polypoidal mass arising from the vestibular region of the lateral nasal wall.* Results*. CT scan and MRI showed highly vascular soft tissue mass occupying the anterior part of the left nostril. Preoperative selective embolization followed by transnasal excision was performed. Histopathological examination confirmed the diagnoses of nasal vestibular angiofibroma.* Conclusion*. Extranasopharyngeal angiofibroma is a very rare pathology. It should be kept in mind as a differential diagnosis with any unilateral nasal vestibular mass causing nasal obstruction and epistaxis. A biopsy without further investigation can cause life threatening bleeding in the patient.

## 1. Introduction

Angiofibroma is a benign tumor, consisting of fibrous tissue with varying degrees of vascularity, characterized by a proliferation of stellate and spindle cells around the blood vessels. It is originating from the pterygoid plate and the region of the sphenopalatine foramen. There are many theories trying to explain the pathology underlying this tumor, like genetic, hormonal, and developmental ones, but none of them had general acceptance. It has been hypothesized that angiofibroma is a testosterone-dependent tumor that arises from a fibrovascular nidus in the nasopharynx that lies dormant until the onset of puberty; hence the incidence is more in males with peak incidence between the ages of 14 and 18 years [1]. Histologically, angiofibroma is made up of fibrous and vascular components, with varying ratio between both of them. Mostly, the vessels are just endothelium-lined spaces without muscle coat, and that accounts for the severe bleeding as the vessels lose the ability to contract [2]. It is the most common vascular neoplasm of the nasal cavity, representing 0.5% of all head and neck tumors [1, 3, 4]. Although it is a benign tumor, it is locally invasive. Progressive nasal obstruction is the most common presenting symptom [5]. Despite the fact that it most commonly arises from the nasopharynx, it may rarely arise in extranasopharyngeal sites, like maxillary sinus which is the most common site (24.6%–32%) [4–7]. Other rare reported sites include ethmoid and sphenoid sinuses, nasal septum, middle turbinate, inferior turbinate, conjunctiva, molar and retromolar region, and tonsil and larynx [1]. Here, we report a very rare case of extranasopharyngeal angiofibroma originating from the lateral wall of the left nasal vestibular area.

## 2. Case Report

**Figure 1:**
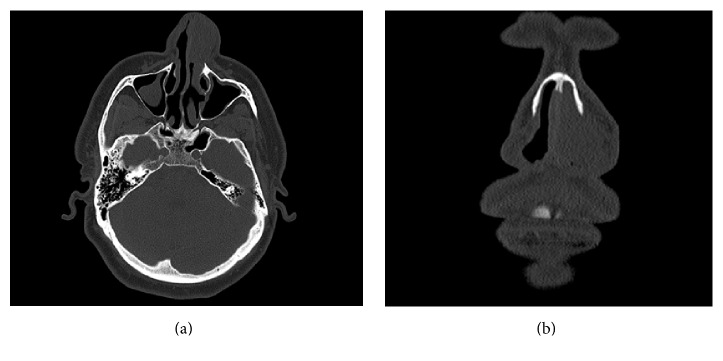
(a) shows axial and (b) shows coronal CT images. Both show a soft tissue mass occupying the left nasal vestibular area.

**Figure 2:**
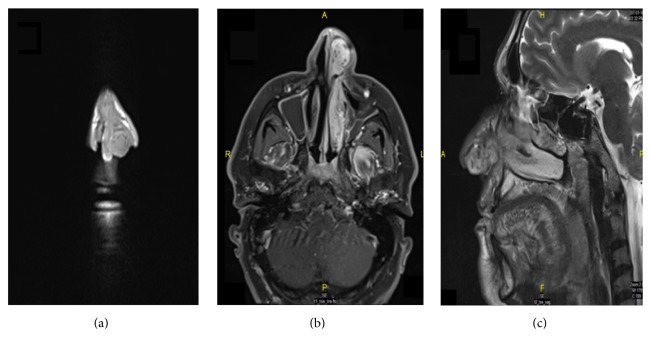
MRI of nose and paranasal sinuses showing a highly vascular mass occupying the left nasal vestibular area. (a) shows T1 coronal view, (b) shows T1 postcontrast axial view, and (c) shows a T2 sagittal view.

**Figure 3:**
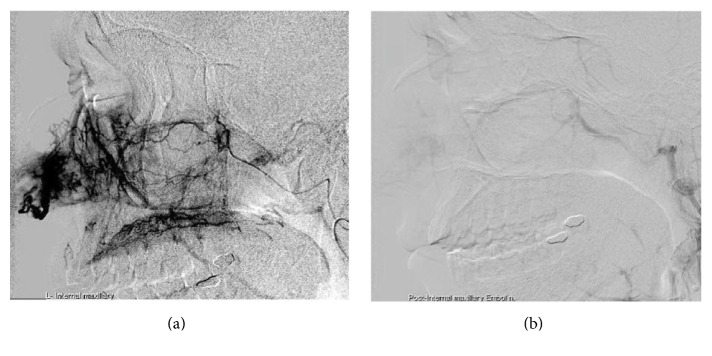
(a) Angiography showing hypervascular nasal mass supplied by the distal branches of the left internal maxillary artery and distal branches of the left facial artery. (b) The mass after embolization of the internal maxillary artery.

A 46-year-old male, not known to have any chronic illness, presented to the emergency department in Hamad Medical Corporation due to one-month history of left side intermittent anterior nasal bleeding increasing in severity and associated with nasal blockage. He had a history of decreased sense of smell and headache for one month. There was no history of trauma, infection, nasal allergies, or bleeding disorders. ENT examination showed mildly deviated nasal septum to the right side with a left sided pinkish colored polypoidal mass originating from the vestibular region of the left lateral nasal wall, easily bleeding upon manipulation. Ears and throat examination was normal. Silver nitrate cauterization was done and bleeding was controlled. So the patient was discharged and he was referred to ORL-HNS clinic for follow-up. The white blood cells count (WBC) was 8.8 × 10^3^/mm^3^, hemoglobin (Hb) level 12.5 g/100 mL, and platelets count (Plt) 278 × 10^3^/mm^3^. Liver and kidney function tests were normal. CT scan of paranasal sinuses showed 3.4 × 2.5 × 1.9 cm mass localized to the anterior part of the left side of the nasal cavity with no extension to the choana or to the paranasal sinuses (Figures 1(a) and 1(b)). MRI (done to rule out hypervascularity of the mass before surgical intervention) showed a hypervascular mass in the same previously described position (Figures 2(a), 2(b), and 2(c)). 24 hours before the surgery, angiography was done which showed hypervascular left side nasal mass supplied by the distal branches of the left internal maxillary artery and distal branches of the left facial artery. Selective embolization was done, to decrease the intraoperative bleeding, with polyvinyl alcohol temporary occlusive particles (PVA), which reduced 85% of the blood flow to the area (Figures 3(a) and 3(b)).

**Figure 4:**
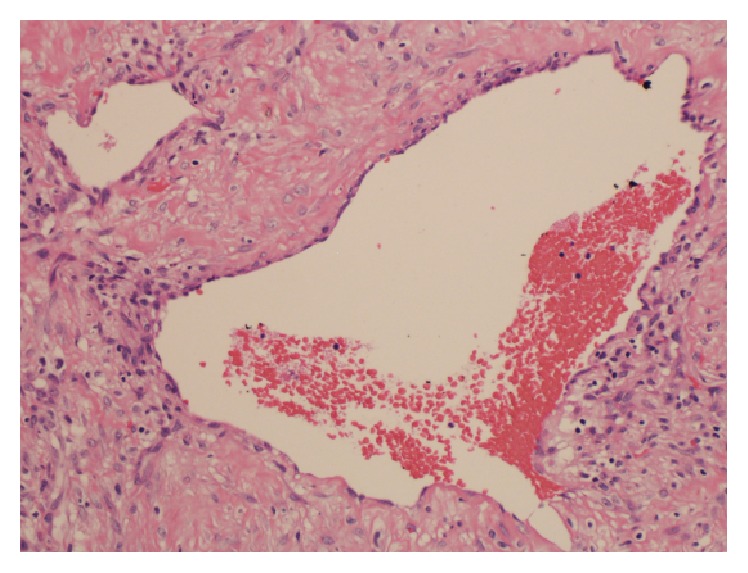
Richly vascular lesion which has variable-sized thin-walled vessels surrounded by a fibroblastic stroma. The vessels have a single endothelial cell lining without a muscularis layer (H&E 20x).

The removal of the tumor was performed under general anesthesia. The pedicle had been completely transected with bipolar diathermy, with insignificant bleeding which was controlled with electrocautery. Nasal pack was inserted in the left nostril only. The postoperative period was uneventful, and the pack was removed on the third postoperative day with no bleeding, and the patient was discharged on the same day. The mass was sent for histopathology. Immunohistochemical staining was performed for AE1/3, vimentin, SMA, desmin, S-100, CD34, CD31, CD117, CD99 BCL-2, and ki-67. The histomorphology and immunohistochemical staining profile supported the diagnosis of angiofibroma (Figure 4). The patient was followed up in the clinic and clinical examination during his last visit (6 months after surgery) showed no recurrence and clear site of operation.

## 3. Discussion

The nasal vestibule is lined with keratinizing squamous epithelium and contains different components such as sebaceous glands and sweat glands. So the pathologic lesions in the nasal vestibule are different from those in the nasal cavity proper; they can be infectious, inflammatory, benign, or malignant tumors. Differential diagnoses can include nasal vestibule cyst, fibroma, squamous papilloma, trichofolliculoma, pseudoepitheliomatous hyperplasia, sebaceous cyst carcinoma, hidradenoma, rhinoscleroma, and malignant melanoma [8, 9]. Here we report a case of nasal vestibule angiofibroma, which is the only hypervascular tumor among all the other mentioned diagnoses.

The presenting symptoms of the nasal vestibular angiofibroma are nasal obstruction and epistaxis [2, 3, 5]. The main differences in clinical presentation of extranasopharyngeal angiofibroma (ENA) versus nasopharyngeal angiofibroma (NA) are the sex predilection, age of presentation, and vascularity. With regards to gender predilection, NA is predominantly a disease of males. However, ENA is of a greater incidence relative to NA in females. Also ENA presents in older age group more than NA, with mean age of 22 years and 17 years, respectively [3, 7], while for nasal vestibule angiofibroma the mean age is 43 years. The administration of contrast agent in NA leads to a strong and usually homogeneous enhancement on CT and MRI, while EN varies from strong to minimal or even no enhancement, due to the frequent poor vascularity of the tumor [10].

The management of any vestibular mass should include preoperative radiological examination. CT scan and MRI (with contrast) are essential for evaluating the extension and vascularity of the lesion. However, signs of suspected hypervascularity, upon CT scan or MRI, indicate the need for angiography with selective embolization prior to any surgical intervention, to reduce the risk of bleeding during the surgery. The use of angiography and selective embolization should have a high threshold, that is, only if there is radiological evidence of diffuse, large, and hypervascular lesion, as these procedures come with their own complications. There are three cases of nasal vestibule angiofibroma previously reported in the literature with no preoperative embolization; all had different amount of intraoperative bleeding varying from minimal to profuse (Table 1). Perhaps smaller lesions can be excised without the need for such elaborate perioperative measures like angiography and embolization. However, the diagnosis should be confirmed by sending the excised lesion for histopathology. The NA have a recurrence rate ranging from 6 to 27.5% [7], while no recurrence was reported in the literature for ENA.

## 4. Conclusion

1. Although extranasopharyngeal angiofibroma is very rare diagnosis, it should be kept in mind as a differential diagnosis with any unilateral nasal vestibular mass causing nasal obstruction and epistaxis.
2. CT scan and MRI are essential in the preoperative evaluation of a vestibular mass to assess for hypervascularity.
3. The use of angiography and selective embolization should have a high threshold, that is, only if there is radiological evidence of diffuse, large, and hypervascular lesions which are predictive factors of intraoperative profuse bleeding.

## Figures and Tables

**Table 1:** Reported cases of vestibular extranasopharyngeal angiofibroma.

Authors	Year	Age	Sex	Location	Symptoms	Onset	Therapy	Pre-op embolization
Kim et al. [8]	2013	56 years	Female	Right nasal vestibule	Progressive swelling	3 years	Endonasal resection (bleeding)	No
Pillenahalli Maheshwarappa et al. [1]	2013	10 years	Male	Left nasal vestibule	Nasal obstruction and epistaxis	4 months	Endoscopic excision (profuse bleeding)	No
Sharanabasappa [11]	2013	60 years	Male	Left nasal vestibule	Nasal mass	1 year	Transnasal resection (minimal bleeding)	No
Present case	2014	46 years	Male	Left nasal vestibule	Recurrent epistaxis and nasal obstruction	1 month	Transnasal resection (insignificant bleeding)	Yes
